# Invariant Natural Killer T Cells Play a Role in Chemotaxis, Complement Activation and Mucus Production in a Mouse Model of Airway Hyperreactivity and Inflammation

**DOI:** 10.1371/journal.pone.0129446

**Published:** 2015-06-12

**Authors:** Piia Karisola, Maili Lehto, Pia Kinaret, Niina Ahonen, Rita Haapakoski, Minna Anthoni, Masaru Taniguchi, Henrik Wolff, Anne Puustinen, Harri Alenius

**Affiliations:** 1 Unit of Systems Toxicology, Finnish Institute of Occupational Health, Helsinki, Finland; 2 RIKEN Center for Integrative Medical Sciences, Laboratory for Immune Regulation, RCAI Kanagawa, Japan; French National Centre for Scientific Research, FRANCE

## Abstract

CD1d-restricted invariant natural killer T (iNKT) cells play a critical role in the induction of airway hyperreactivity (AHR). After intranasal alpha-galactosylceramide (α-GalCer) administration, bronchoalveolar lavage fluid (BALF) proteins from mouse lung were resolved by two-dimensional differential gel electrophoresis (2D-DIGE), and identified by tandem mass spectroscopy. A lack of iNKT cells prevented the development of airway responses including AHR, neutrophilia and the production of the proinflammatory cytokines in lungs. Differentially abundant proteins in the BALF proteome of α-GalCer-treated wild type mice included lungkine (CXCL15), pulmonary surfactant-associated protein D (SFTPD), calcium-activated chloride channel regulator 1 (CLCA1), fragments of complement 3, chitinase 3-like proteins 1 (CH3LI) and 3 (CH3L3) and neutrophil gelatinase-associated lipocalin (NGAL). These proteins may contribute to iNKT regulated AHR via several mechanisms: altering leukocyte chemotaxis, increasing airway mucus production and possibly via complement activation.

## Introduction

Asthma affects 5–10% of the population in the developed countries and represents a huge socioeconomic burden [1]. This complex and heterogeneous inflammatory disease is characterized by bronchial hyperresponsiveness, mucus hypersecretion and airway inflammation [2]. Conventional CD4^+^ T cells recognize protein antigens, infiltrate into the lungs, produce several Th2-type cytokines such as IL-4, IL-5 and IL-13, and orchestrate the lung inflammation. Th2-responses are important during the development of asthma, although it seems that they alone are not sufficient to induce and maintain AHR and inflammation in the airways [3, 4]. Th2-based strategies have failed to offer any major benefits in the treatment of asthma, especially in the modulation of airway hyperresponsiveness, and clearly there is an urgent need to identify additional targets and biomarkers.

CD1d-restricted invariant natural killer T (iNKT) cells, which also express CD4 on their cell surfaces, may play a crucial role in the development of airway hyperreactivity (AHR). INKT cells possess characteristics of both NK cells and conventional T cells, and when activated, they rapidly produce large quantities of many cytokines, including IL-4, IFN-γ and IL-17, which influence adaptive immune responses and change the polarization of conventional T cells. INKT cells use their invariant Vα14-Jα18 T cell receptors to recognize endogenous glycolipid ligand such as isoglobotrihexosylceramide (iGb3) and its synthetic homolog alpha-galactosylceramide (α-GalCer) which are presented on the major histocompatibility complex class I-like protein CD1d on the antigen presenting cells [5]. In a mouse model where administration of α-GalCer induced AHR and inflammation, it was noted that when iNKT cells were absent, AHR has failed to develop although Th2 responses, including lung inflammation, proceeded normally [6].

The proteome is versatile and constantly changing in response to state-dependent protein expression and different protein modifications such as phosphorylation, acetylation, glycosylation and methylation. Proteomics is the only global protein profiling technique compatible with biological fluids such as bronchoalveolar lavage fluid (BALF), which contains a variety of cellular and extracellular protein components originating from the epithelial lining fluid of the bronchus and alveoli. Distinct changes in certain cytokines, such as IL-5 and IL-13, in BALF have been correlated with the extent of allergic airway inflammation [7]. The introduction of proteomics has led to the discovery of proteins that had not previously been associated with a particular disease [8, 9].

In this study, we investigated the role of iNKT cells in the development of α-GalCer-induced AHR and airway reactivity and identified proteins secreted into BALF during airway responsiveness in wild type (WT) mice as compared to CD1d^-/-^ and to Jα18^-/-^ mice. The results reveal that iNKT cells orchestrate the induction of leukocyte chemotaxis, complement activation, and airway mucus production, and these phenomena are missing in vehicle-treated WT mice or in iNKT cell deficient mice after α-GalCer administration.

## Materials and Methods

### Mice

Female BALB/c mice were purchased (6–8 weeks old) from Scanbur AB (Sollentuna, Sweden), CD1d^-/-^ mice from The Jackson Laboratories (Bar Harbor, Maine, USA) and Jα18^-/-^ mice were a kind gift from Prof. Taniguchi [10], both strains are developed against a BALB/C background. Mice were housed in cages, provided with food and water ad libitum, and maintained on a 12/12-h light/dark cycle. The experiments were conformed to the European Convention for the Protection of Vertebrate Animals Used for Experimental and Other Scientific Purposes (Strasbourg March 18, 1986, adopted in Finland May 31, 1990). The study was approved by the Animal Experiment Board and the State Provincial Office of Southern Finland (decision STH94A).

### Treatment protocols

BALB/c, CD1d^-/-^ or Jα18^-/-^ mice were anesthetised with isoflurane and given DMSO (Sigma) vehicle or 1 μg of α-GalCer (Alexis corp., Lausen, Switzerland; dissolved in DMSO) intranasally (i.n.) in a 50 μl volume. The airway resistance and compliance were measured from sensitized and challenged mice as previously described [11].

### Determination of Airway Reactivity to Methacholine

At 24 hours after the α-GalCer administration, responses to inhaled methacholine (Sigma-Aldrich, St. Louis, MO) were measured in conscious, unrestrained mice using whole-body plethysmography (WBP; Buxco Research Systems, Wilmington, NC). AHR was expressed as enhanced pause values, as described in detail previously [12]. In the invasive measurement of lung resistance (R_L_), mice were anesthetized with Hypnorm/Dormicum solution (10 ml/kg; VetaPharma, Leeds, UK) and their tracheas were cannulated. Mice were placed in a resistance/compliance (RC) collection station (FinePointe RC; Buxco Research Systems, Winchester, UK), and were mechanically ventilated at a rate of 150 breaths/min. PBS and increasing doses of aerosolized methacholine (3, 6, 12, 24, and 48 mg/ml) were administered via the trachea, and changes in R_L_ were expressed as a mean value of 3 minutes of recording.

### Bronchoalveolar Lavage Fluid (BALF)

BALF was collected 24 hours after the intranasal administration by flushing the lungs with 0.8 ml of PBS. Differential cell counts were counted from May-Grünwald-Giemsa-stained (MGG) cytospin preparations in 20 high-power fields under light microscopy (Leica DM 4000B; Leica, Wetzlar, Germany). Part of the left lung was removed for RNA isolation, and another part embedded in OCT (Sakura Finetek, Tokyo, Japan) on dry ice prior to immunohistological evaluation.

### Histology and immunohistochemisty

In the immunohistochemistry, 4-μm cryostat sections from a lung were prepared, air dried, fixed in acetone (-20°C) for 10 minutes, and stored at -80°C. All primary Abs (anti-mouse CD4 and anti-mouse CD8) and isotype controls were purchased from BD Pharmingen (San Diego, CA). Biotin-conjugated secondary Ab anti-rat IgG (heavy and light chains) was purchased from Vector Laboratories (Burlingame, CA).

### RT-PCR analysis

Total RNA extractions with Trisure Reagent (Bioline, London, UK), complementary DNA synthesis, and real-time PCR (RT-PCR) were performed as previously described [13].

### 2D-DIGE and DeCyder analysis

Collected BAL samples were centrifuged (10 min, 800 x g, at +4°C) to remove cells. BAL supernatants from two mice were pooled to provide enough protein for 2D-DIGE analysis. The samples were concentrated by ultrafiltration (Amicon Ultra-15 5000 MWCO, Millipore, Ireland) to 120 μL. After the determination of the protein concentration (DC Protein Assay, Bio-Rad) BALF proteins were precipitated with 2-D Clean-Up Kit (GE Healthcare) and dissolved to a protein concentration of 2 μg/μl in 7 M urea, 2 M thiourea, 4% CHAPS (Anatrace, Maumee, OH, USA) and 30 mM Tris-HCl, pH 8.8. Then, 10 μl from each sample was pooled for the internal standard and 40 μg of BALF proteins were then labelled using 200 pmol Cy3 or Cy5 dyes (CyDye DIGE Fluor minimal dyes; GE Healthcare) according to the Ettan 2-D DIGE instructions. Cy2Dye was used as the internal standard whereas Cy3 and Cy5 labellings were randomized evenly between the study groups. A total of 40 μg of Cy2-labelled internal control sample was added for to each combined Cy3- and Cy5-labelled sample pair in the quantitative 2D-DIGE analysis.

CyDye labelled protein samples (four in each study group) were separated by isoelectric focusing (IEF) using IPG strips (18 cm, pH 3–10, GE Healthcare). The IEF strips were rehydrated for 6 hours in 340 μL of the rehydration solution (7 M urea, 2 M thiourea, 4% CHAPS, 0.04% bromophenol blue (BPB) containing 0.5% IPG buffer, pH 3–10NL (GE Healthcare) and 1.2% DeStreak reagent (GE Healthcare). The samples were absorbed onto the IPG strips by cup-loading. IEF was performed using Ettan IPGphor II (GE Healthcare) at +20°C using a limit of 75 μA/strip as follows: 3 hrs at 150 V, 2 hrs at 300 V, a linear gradient from 300 V to 1000 V for 6 hrs, the 2nd gradient from 1000 V to 8000 V for 2 hrs and finally 2 hrs at 8000 V (total 29 000 Vhrs). The strips were stored at -20°C. Just before undergoing SDS-PAGE, the strips were thawed at room temperature and incubated first for 15 min in a buffer containing 50 mM Tris–HCl (pH 6.8), 6 M urea, 2% SDS, 0.04% BPB and 30% glycerol with 1% dithiotreitol (DTT), followed by a 15 min equilibration in the same buffer with 2% iodoacetamide instead of DTT. The second dimension SDS-PAGE was run on 12.5% gels with the Ettan DALTSix System (GE Healthcare): 3 W for 1 h and 90 W for 4 h (six gels).

Gels were scanned between low fluorescence glass plates using an Ettan DIGE Imager (GE Healthcare) at wavelengths of 480 nm for Cy2, 540 nm for Cy3 and 680 nm for Cy5 with 100 μm pixel size. After scanning, the gels were fixed in 30% ethanol, 1% acetic acid and silver stained. The cropped images of 2-D DIGE gels were analyzed for protein expression differences using DeCyder 2D 7.0 software (GE Healthcare).

### Identification of protein spots

Differentially expressed protein spots were in-gel digested as previously described [14]. In brief, the protein spots were excised from the gels, reduced with DTT, and alkylated with iodoacetamide before in-gel digestion with trypsin (modified sequencing grade porcine trypsin, 0.04 μg/μl, Promega, Madison, WI, USA) for 16 h at +37°C. After removing supernatants to fresh tubes, the remaining peptides were extracted twice from the gel pieces by using 100 μl of 5% formic acid in 50% acetonitrile (ACN). The extracts were then dried in a vacuum centrifuge, and dissolved in 2% formic acid. Each peptide mixture was analyzed with an automated nanoflow capillary LC–MS/MS using CapLC system (Waters, Milford, MS, USA) coupled to an electronspray ionization quadrupole time-of-flight (Q-TOF) mass spectrometer (Waters). Reversed phase separations were accomplished with a 75 μm × 15 cm NanoEase Atlantis dC18 column at a flow rate of 200 nl/min. Solvent A was 0.1% formic acid in 5% ACN, and solvent B 0.1% formic acid in 95% ACN. The peptide separation was achieved with a linear gradient of 0–60% of solvent B in 30 min.

The obtained mass fragment spectra were analyzed with in-house Mascot v.2.1 (Matrix Science Ltd., London, UK) and searched against human or mammalian entries in the NCBInr database. One missed cleavage was allowed, and searches were performed with fixed carbamidomethylation of cysteine residues, and variable oxidation of methionine, histidine, and tryptophan residues. A peptide tolerance of 0.2 Da and fragment tolerance of 0.5 Da were used with trypsin as the specified digestion enzyme. A minimum number of two matched peptides or a Mascot score higher than 70 was considered significant.

### Bioinformatics

In the bioinformatics analyses, the intensity values of protein spots after Cy2 standardization were imported to the R Software, version 3.0.0 (http://cran.r-project.org). Hierarchical clustering in a heatmap was performed using the complete linkage method with the Manhattan distance measure. In the VENN-diagram, the Limma package [15] was used for linear model fitting and empirical Bayes pairwise comparisons made between the α-GalCer-treated groups of WT, Jα18^-/-^ and CD1d^-/-^ and WT vehicle-treated group. Proteins with nominal p-value <0.01 after Benjamini Hochberg post hoc correction were assessed as significant and applied to the VENN-diagram. DAVID bioinformatics resources (http://david.abcc.ncifcrf.gov/) were used to identify enriched protein functional annotations, gene ontology terms or pathways [16].

### Western blot

BALF samples (7.5 μl) were run on 10–20% Tris-HCl Criterion Precast gels (26 wells, comb 15 μl, Bio-Rad Laboratories, Hercules, CA, USA) at 100 V for 5–10 minutes, and 200 V for 60 minutes in running buffer (Bio-Rad) of 25 mM Tris 192 mM glycine and 0.1% SDS, pH 8.3. A pool of all BALF samples was used as an internal standard on each gel to provide a reference for normalization of the results. Dual Color (Bio-Rad) and Magic Mark (Invitrogen, Carlsbad, CA, USA), were used as molecular weight markers. Proteins were transferred to polyvinylidene difluoride membranes (Immobilon-P, Millipore, Billerica, MA, USA) using the Criterion Blotter (Bio-Rad) at 300 mA for 2h in transfer buffer (25 mM Tris, 192 mM glycine, 20% methanol). The membranes were blocked with 0.05% Tween 20 in PBS, and incubated in a shaker at +4°C overnight with polyclonal antibodies to complement C3/C3a (C3C3a, anti-mouse chicken IgY, diluted 1:2000) from Abcam (Cambridge, UK) and chloride channel calcium activated 3 (CLCA1, anti-mouse rabbit IgG, diluted 1:1000) from Abcam and with monoclonal primary antibody to lungkine (CXCL15, anti-mouse rat IgG2B, diluted 1:1000) from R&D Systems (Minneapolis, MN, USA). Immunoblots were then incubated with polyclonal peroxidase-conjugated immunoglobulins; rabbit anti-rat and goat anti-rabbit (Dako, Glostrup, Denmark, diluted 1:2000) or rabbit anti-chicken (Abcam, 1:20 000) at room temperature for 1 h. The proteins were visualized with the Plus ECL detection kit (Perkin Elmer Inc., Waltham, MA, USA). The blots were imaged with ImageQuant LAS 4000 mini, software version 1.0 (GE Healthcare, Piscataway, NJ, USA). The 1D gel analysis and quantitation of the protein bands were accomplished by calculating the intensities of the bands with image analysis software ImageQuant TL v.7.0 from GE Healthcare. Statistical analyses for Western blots were performed using GraphPadPrism 5 software (v.5 GraphPad Software, La Jolla, CA).

### Statistics

Single-group comparisons were made using the nonparametric Mann-Whitney U-test. Results are expressed as mean ±SEM and P-values of <0.05 were considered statistically significant. Statistical analyses were performed using GraphPadPrism (v.5 GraphPad Software, La Jolla, CA).

## Results

### A-GalCer induces severe AHR and airway inflammation

**Fig 1:**
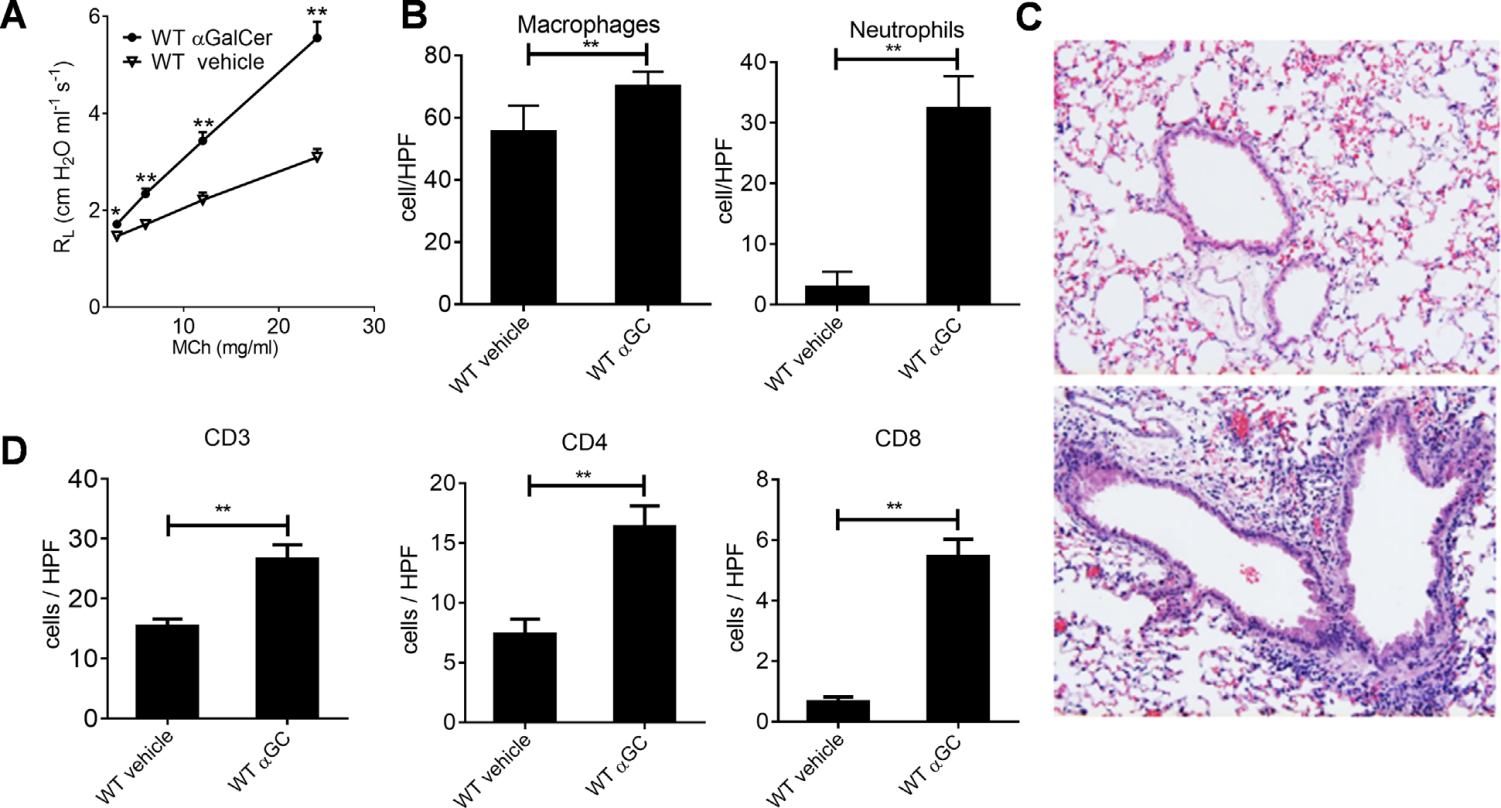
A-GalCer induces AHR, neutrophil influx and inflammation in lungs of WT mice. A. Direct lung resistance (R_L_) was measured in WT mice after 24 hours after i.n. administration of vehicle or α-GalCer (αGC) using the ELAN system. B. Macrophage and neutrophil cell distributions were calculated (20x magnification) from cytospinned and MGG-stained BALF samples. C. Vehicle (upper figure) and α-GalCer—treated (lower figure), paraffin embedded lung sections were H&E stained, and D. Corresponding cryosections were stained with anti-CD3, anti-CD4 or with anti-CD8 mAbs to identify the number and localization of different T cell subsets. *P, 0.05; **P, 0.01; ***P, 0.001. n = 8 mice/group.

To investigate the effects of α-GalCer, BALB/c mice were intranasally exposed to α-GalCer or to vehicle, and their lung function was measured 24 hours later. Lung resistance (R_L_) to methacholine was clearly enhanced in the α-GalCer-treated group as compared to the vehicle-treated group (Fig 1A). The numbers of macrophages and neutrophils in BALF were significantly increased after α-GalCer treatment, while there still were no eosinophils or lymphocytes (Fig 1B and S1 Fig). In addition, the extent of the inflammation around the bronchioles was enhanced (Fig 1C), whereas in iNKT cell deficient mice there were no signs of inflammation in lung tissue (S1 Fig). The numbers of CD3^+^ T cells, including both CD4^+^ and CD8^+^ cells, were also highly increased in α-GalCer-treated mice when compared to vehicle-treated mice (Fig 1D).

### Lack of iNKT cells attenuates AHR and inflammation

**Fig 2:**
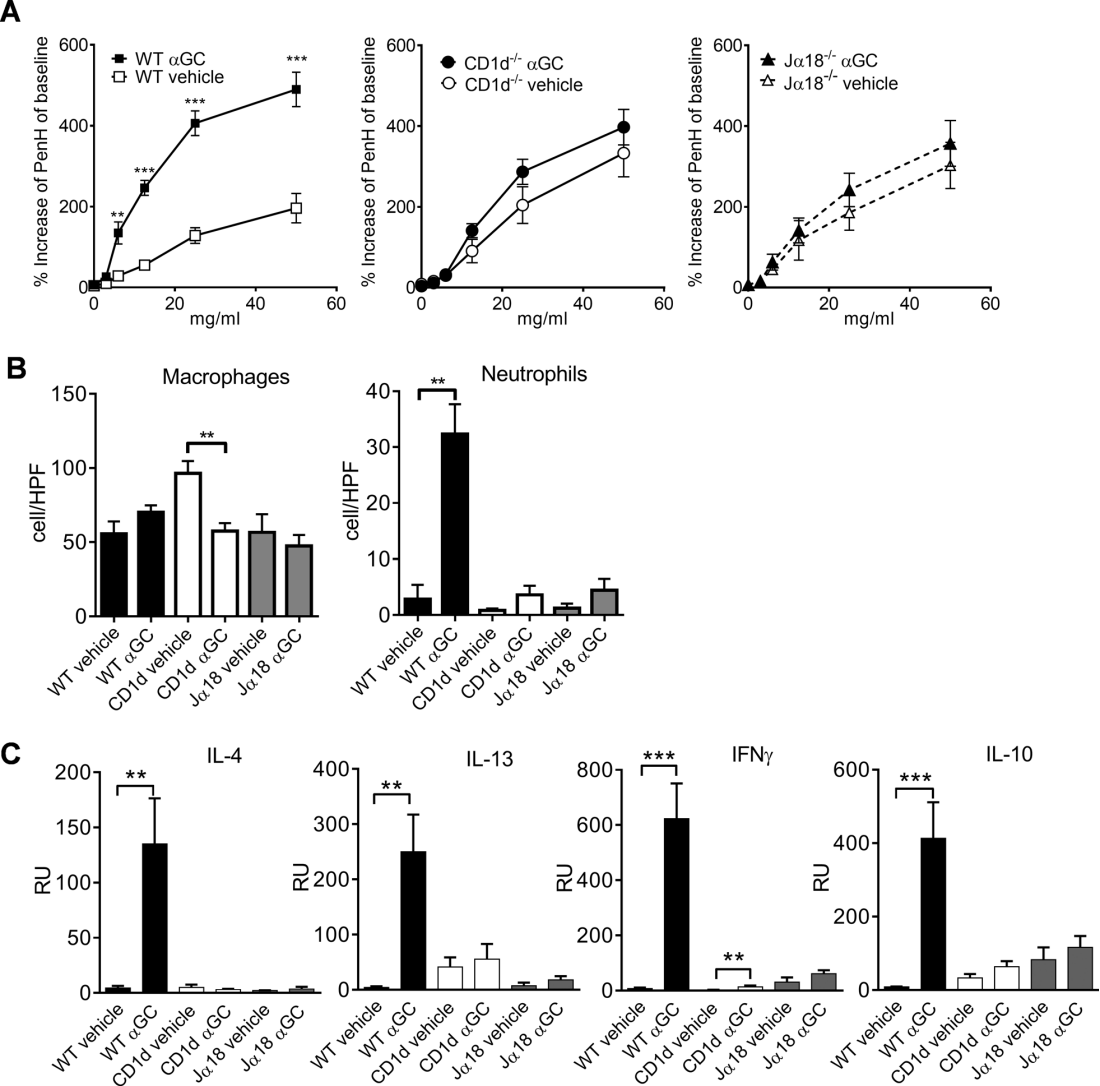
NKT-cell-deficient mice have attenuated AHR, neutrophil influx and cytokine production as compared to WT mice. A. AHR measured in WT, CD1d^-/-^ and in Jα18^-/-^ mice after 24 hours after i.n. administration of α-GalCer (αGC) using BUXCO system. B. Macrophage and neutrophil cell distributions of cytospinned and MGG-stained BALF samples, and C. mRNA expression of IL-4, IFN-γ, IL-10 and IL-13 cytokines in lung samples by TaqMan RT-pcr. *P, 0.05; **P, 0.01; ***P, 0.001. n = 8 mice/group.

To study how iNKT cells contribute to the development of AHR and inflammatory responses in the respiratory tract, we administered α-GalCer to the mouse lungs in the presence or absence of iNKT cells. AHR did not develop in CD1d^-/-^ and Jα18^-/-^ mouse strains, which lack iNKT cells (Fig 2A). In the control group, CD1d-deficient mice had more macrophages in their BALF than the other strains but their amount decreased when treated with α-GalCer (Fig 2B). In contrast, only α-GalCer-treated WT mice displayed a significant increase in the numbers of neutrophils in BALF, this response was absent in both iNKT cell deficient strains (Fig 2B).

We next examined whether iNKT cells are required for the development of the Th2-type environment in the lungs. Only in the WT mice, did α-GalCer increase the production of the Th2-type cytokines IL-4 and IL-13, Th1-type IFN-γ and regulatory IL-10 and again, this effect was not present in the iNKT cell deficient mice (Fig 2C).

### Proinflammatory proteins are identified in the 2D gels

**Fig 3:**
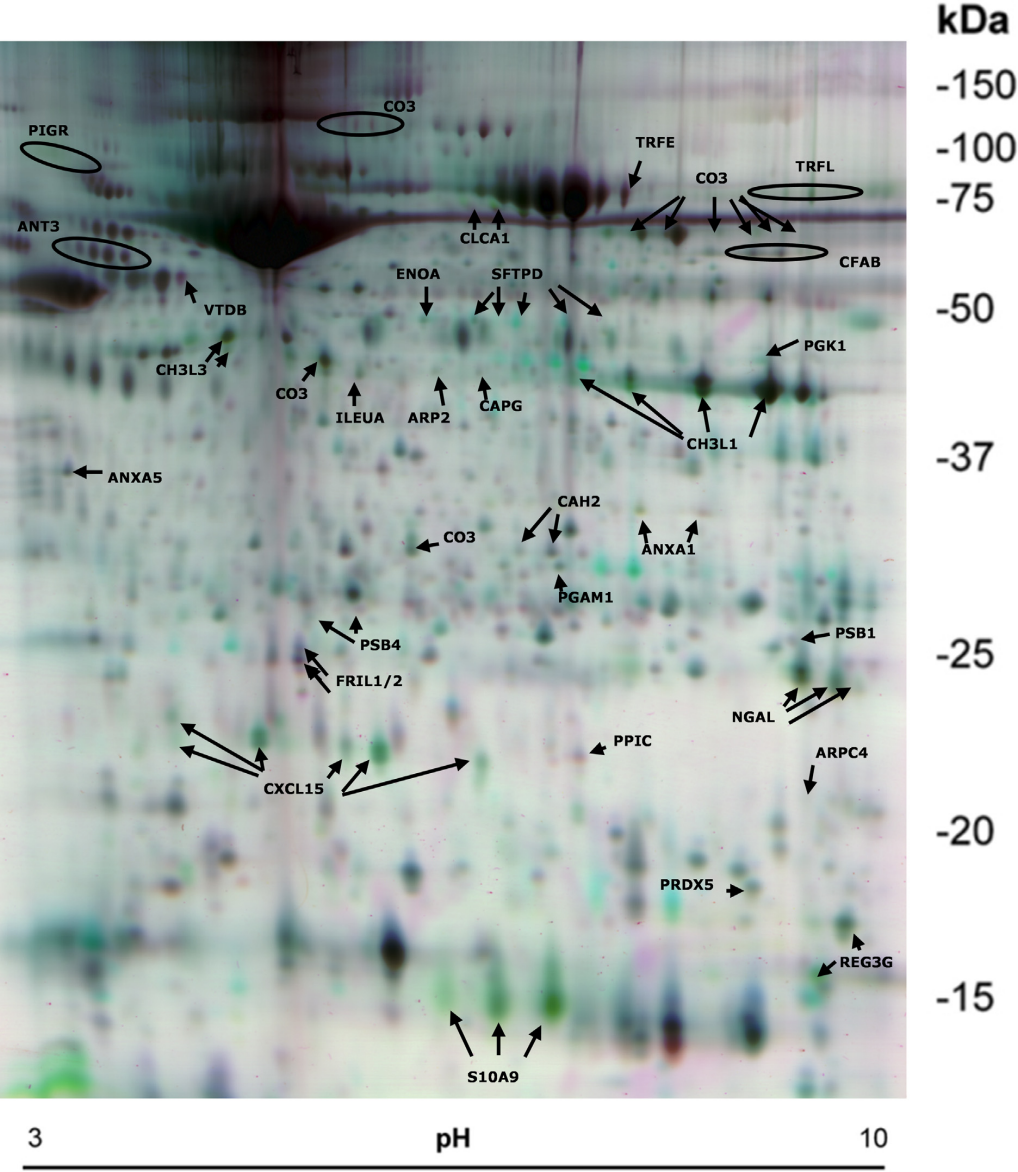
False color 2D-DIGE image of BALF protein spots. WT BALB/c, CD1d^-/-^ and Jα18^-/-^ mice were exposed to α-GalCer or to a vehicle and their BALFs were used for 2D-DIGE. In the shown gel pair, purple spots represent decreased abundance and green spots represent increased protein abundance in the α-GalCer—treated WT samples as compared to vehicle-treated WT samples. Protein spots identified with LC-MS/MS are labelled with their UniProt entry name and are listed in Table 1.

To assess the mechanisms of α-GalCer-mediated airway responses in more detail, we conducted a proteomic analyses of BALF from vehicle and α-GalCer-treated WT, CD1d^-/-^ and Jα18^-/-^ mice. BALF samples were obtained 24 hours after the exposure, at the time when AHR is present, but inflammation, mucosal remodeling and cytokine production are still evolving. Similar protein patterns were observed in the 2D gels of all vehicle-treated groups (Fig 3, and data not shown). Image analysis revealed about 2000 protein spots which were consistently detected and matched on all 24 separate gels of each treatment group (vehicle or α-GalCer). Table 1 summarizes the identified 74 differentially abundant protein spots for α-GalCer-treated-WT mice in comparison with the α-GalCer-treated Jα18^-/-^ and CD1d^-/-^, and to vehicle-treated WT samples.

**Table 1:** List of identified proteins.

**Increased abundance in a-GalCer-treated WT**									
**UniProt Entry**	**Av. Ratio**	**T-test**	**1-ANOVA**	**Protein Name**	**UniProt AC**	**Mass (kD)^a)^**	**Mascot Sc.**	**Peptides**	**Coverage (%)^b)^**
CXL15	4,58	3,70E-12	5,27E-11	C-X-C motif chemokine 15	Q9WVL7	19	159	5	28
CXL15	2,25	3,70E-12	7,26E-11	C-X-C motif chemokine 15	Q9WVL7	19	89	2	9
CXL15	2,86	5,13E-11	5,27E-11	C-X-C motif chemokine 15	Q9WVL7	19	109	4	20
PIGR	2,96	2,34E-10	2,44E-10	Polymeric immunoglobulin receptor	O70570	66 (85)	109	2	3 (2)
CXL15	2,44	3,24E-10	2,79E-09	C-X-C motif chemokine 15	Q9WVL7	19	104	3	16
SFTPD	1,99	3,24E-10	1,08E-08	Pulmonary surfactant-associated protein D	P50404	37,5	323	5	14
PIGR	2,61	4,81E-10	2,44E-10	Polymeric immunoglobulin receptor	O70570	66 (85)	476	8	12 (9)
S10A9	7,82	6,11E-10	3,82E-09	Protein S100-A9 = Calgranulin B	P31725	13	293	6	46
PIGR	2,8	6,11E-10	7,26E-11	Polymeric immunoglobulin receptor	O70570	66 (85)	543	9	14 (11)
SFTPD	2,58	6,11E-10	2,72E-09	Pulmonary surfactant-associated protein D	P50404	37,5	149	3	9
ANXA1	5,71	6,57E-10	2,02E-08	Annexin A1	P10107	38,6	127	3	7
TRFL	2,73	1,10E-09	5,47E-08	Lactotransferrin	P08071	77	151	3	3
SFTPD	2,34	1,10E-09	3,82E-09	Pulmonary surfactant-associated protein D	P50404	37,5	84	3	8
S10A9	6,12	4,17E-09	3,99E-08	Protein S100-A9 = Calgranulin B	P31725	13	210	4	37
REG3G	2,81	4,17E-09	2,44E-10	Regenerating islet-derived protein 3-gamma 15 kD	O09049	15	61	2	20
CH3L1	2,09	4,17E-09	1,58E-07	Chitinase-3-like protein 1	Q61362	43	338	9	22
CXL15	2,06	5,07E-09	1,67E-07	C-X-C motif chemokine 15	Q9WVL7	19	138	4	20
CO3	1,65	5,56E-09	5,47E-08	Complement C3 beta chain	P01027	72 (186)	87	1	2(0)
CO3	2,73	6,02E-09	2,15E-07	Complement C3 beta chain	P01027	72 (186)	950	20	36 (14)
S10A9	5,77	6,43E-09	2,15E-07	Protein S100-A9 = Calgranulin B	P31725	13	264	5	38
TRFL	2,13	8,21E-09	7,10E-08	Lactotransferrin	P08071	77	717	17	22
CAPG	1,5	9,41E-09	3,79E-07	Macrophage-capping protein	P24452	39,2	518	10	32
CXL15	1,73	9,73E-09	8,51E-08	C-X-C motif chemokine 15	Q9WVL7	19	107	3	16
PIGR	2,23	1,29E-08	1,14E-08	Polymeric immunoglobulin receptor	O70570	66 (85)	578	12	21 (16)
CH3L1	1,67	1,31E-08	4,39E-07	Chitinase-3-like protein 1	Q61362	43	477	11	28
CO3	2,15	1,49E-08	3,89E-07	Complement C3c alpha' chain fragment 2	P01027	38 (186)	639	13	38 (8)
PSB1	1,88	1,49E-08	1,47E-08	Proteasome subunit beta type-1	O09061	23,6	320	10	47
NGAL	2,39	1,59E-08	8,83E-08	Neutrophil gelatinase-associated lipocalin	P11672	22,9	142	4	15
CO3	2,28	1,85E-08	5,97E-07	Complement C3 beta chain	P01027	72 (186)	812	19	33 (13)
ILEUA	2,2	2,42E-08	8,64E-07	Leukocyte elastase inhibitor A	Q9D154	42,5	584	13	43
NGAL	1,92	2,88E-08	1,06E-07	Neutrophil gelatinase-associated lipocalin	P11672	22,9	126	3	13
CH3L3	2,17	3,12E-08	2,02E-07	Chitinase-3-like protein 3	O35744	44	636	12	35
SFTPD	1,61	3,18E-08	7,09E-07	Pulmonary surfactant-associated protein D	P50404	37,5	187	5	14
ARP2	1,98	4,17E-08	5,97E-07	Actin-related protein 2	P61161	44,7	337	7	21
CO3	1,73	4,72E-08	3,80E-07	Complement C3 beta chain	P01027	72 (186)	1914	37	61 (24)
PGK1	1,5	4,72E-08	5,96E-07	Phosphoglycerate kinase 1	P09411	44,5	110	4	11
PIGR	2,24	6,02E-08	2,17E-08	Polymeric immunoglobulin receptor	O70570	66 (85)	533	12	22 (17)
REG3G	1,9	1,34E-07	7,09E-07	Regenerating islet-derived protein 3-gamma 16,5 kD	O09049	16,5	83	1	8
TRFL	2,03	1,41E-07	5,75E-07	Lactotransferrin	P08071	77	269	9	13
CLCA1	1,95	1,41E-07	2,34E-06	Calcium-activated chloride channel regulator 1	Q9D7Z6	75 (100)	1512	26	40 (30)
CH3L1	1,98	1,43E-07	1,02E-06	Chitinase-3-like protein 1	Q61362	43	724	16	39
PRDX5	1,89	1,51E-07	5,33E-07	Peroxiredoxin-5	P99029	21,9	97	2	11
CO3	2	1,54E-07	4,41E-06	Complement C3c alpha' chain fragment 1	P01027	23 (186)	360	10	40 (5)
CH3L3	1,75	2,29E-07	2,15E-07	Chitinase-3-like protein 3	O35744	44	387	11	22
CH3L1	1,55	2,64E-07	5,36E-06	Chitinase-3-like protein 1	Q61362	43	606	14	38
TRFL	2,73	3,40E-07	1,11E-05	Lactotransferrin	P08071	77	71	3	3
TRFL	2,14	4,33E-07	1,09E-05	Lactotransferrin	P08071	77	117	5	6
TRFL	1,89	5,25E-07	1,11E-05	Lactotransferrin	P08071	77	174	6	7
CFAB	1,57	8,62E-07	1,26E-05	Complement factor B	P04186	85	115	4	7
NGAL	2,07	1,02E-06	1,42E-05	Neutrophil gelatinase-associated lipocalin	P11672	22,9	291	6	31
CFAB	1,84	1,52E-06	3,96E-05	Complement factor B	P04186	85	351	9	21
CLCA1	1,75	1,65E-06	1,57E-05	Calcium-activated chloride channel regulator 1	Q9D7Z6	75 (100)	572	13	37 (28)
CO3	1,73	1,65E-06	3,96E-05	Complement C3 beta chain	P01027	72 (186)	269	8	13 (5)
ENOA	1,52	2,34E-06	2,22E-06	Alpha-enolase	P17182	47,1	351	9	21
SFTPD	1,95	4,12E-06	1,09E-05	Pulmonary surfactant-associated protein D	P50404	37,5	176	5	14
CFAB	1,53	5,73E-06	1,27E-04	Complement factor B	P04186	85	151	4	5
PGAM1	1,62	6,00E-06	6,95E-06	Phosphoglycerate mutase 1	Q9DBJ1	28,8	175	5	25
ARPC4	2,1	7,66E-06	1,29E-04	Actin-related protein 2/3 complex subunit 4	P59999	19,5	167	5	23
CFAB	1,64	2,74E-05	4,27E-04	Complement factor B	P04186	85	245	6	13 (9)
CHA2	1,8	4,18E-04	5,97E-07	Carbonic anhydrase 2, down in cd1d	P00920	29	86	2	7
ANXA1	1,66	4,41E-04	9,65E-05	Annexin A1	P10107	38,6	99	3	7
CH3L1	1,5	1,07E-03	2,06E-03	Chitinase-3-like protein 1	Q61362	43	428	10	28
**Decreased abundance in a-GalCer-treated WT**									
**UniProt Entry**	**Av. Ratio**	**T-test**	**1-ANOVA**	**Protein Name**	**UniProt AC**	**Mass (kD)^a)^**	**Mascot Sc.**	**Peptides**	**Coverage (%)^b)^**
FRIL1/2	-2,08	2,64E-08	7,09E-07	Ferritin light chain 1 and 2	P29391/P49945	20,8	720	12	68
ANT3	-1,52	5,18E-08	1,02E-06	Antithrombin III	P32261	52	1201	25	44
ANT3	-1,51	1,61E-07	2,38E-06	Antithrombin III	P32261	52	1183	24	44
TRFE	-1,56	2,47E-07	6,21E-06	Serotransferrin	Q921I1	76,7	803	20	29
PPIC	-1,61	6,42E-07	1,26E-05	Peptidyl-prolyl cis-trans isomerase C	P30412	22,7	142	3	16
PSB4	-1,6	9,53E-07	5,36E-06	Proteasome subunit beta type-4	P99026	29	111	4	10
FRIL1/2	-1,72	2,88E-05	4,50E-04	Ferritin light chain 1 and 2	P29391/P49945	20,8	521	10	66
PSB4	-1,6	7,55E-05	1,36E-04	Proteasome subunit beta type-4	P99026	29	706	11	54
ANT3	-1,5	2,97E-04	1,47E-03	Antithrombin III	P32261	52	711	13	27
CHA2	-1,8	3,99E-04	7,56E-04	Carbonic anhydrase 2	P00920	29	55	3	12
ANXA5	-1,56	5,81E-04	4,53E-03	Annexin A5	P48036	35,7	971	16	56
VTDB	-1,53	7,47E-04	2,67E-04	Vitamin D-binding protein	P21614	53,6	762	15	27

Report of all 74 identified proteins with the information of their UniProt entry name (UniProt) and protein number (UniProt AC). Student’s *t*-test (T-test) and 1-way ANOVA (1-ANOVA) values of the spot and fold ratio of the abundance change between α-GalCer-treated WT samples and the other three (α-GalCer-treated Jα18^-/-^ and CD1d^-/-^, and vehicle-treated WT) mice samples from the DeCyder analysis are reported. Protein score represents the confidence of the identification obtained from Mascot-software.

^a^) In parenthesis is the unprocessed molecular weight of the protein.

^b^) In parenthesis is the sequence coverage of the unprocessed protein.

A marked increase (p<0.001) was observed in some proinflammatory proteins in BALF in α-GalCer-treated WT mice such as neutrophil recruiting lungkine (CXCL15), surfactant—associated protein D (SFTPD), complement factor C3 cleavage products C3 alpha and beta, and chitinase-3-like-1 (CH3L1 or BRP39) and chitinase-3-like-3 (CH3L3 or Ym1). Several proteins, including CXCL15 and CH3L1, existed in multiple spots of similar molecular weights but with different pI values, which may be due to posttranslational modifications. There were increases in the abundance of chloride channel calcium activated 3 protein (CLCA1) and in lesser amounts also in the production of neutrophil gelatin-associated lipocalin (NGAL).

### A-GalCer—treated WT mice have a unique protein expression profile

**Fig 4:**
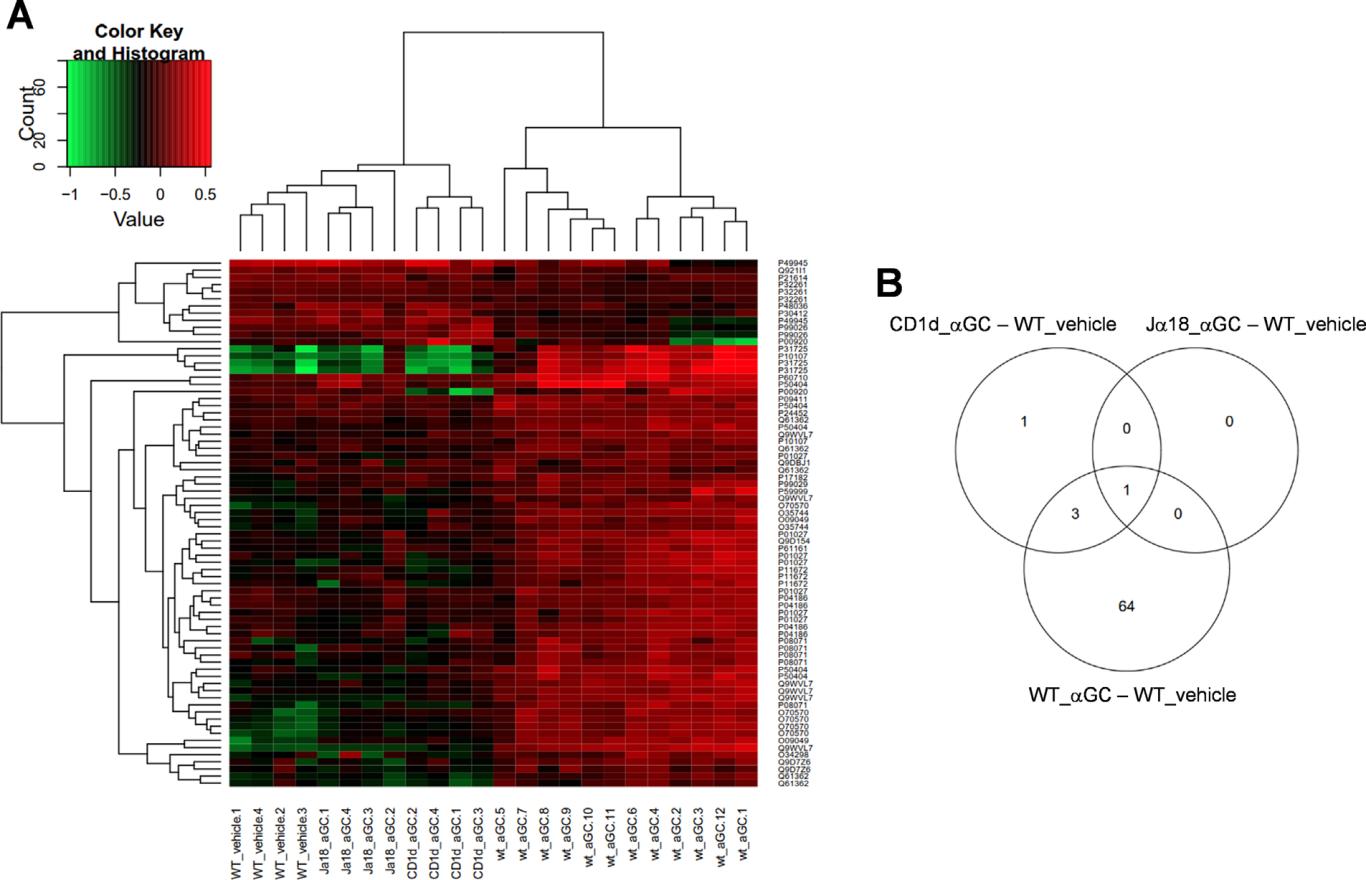
Hierarchial clustering and VENN-diagram of identified proteins. A. Hierarchical clustering of protein spots in groups. Two main clusters are found; one consisting of 12 α-GalCer-treated WT samples, the other containing 8 α-GalCer-treated Jα18-KO and CD1d-KO mice samples and 4 vehicle-treated WT samples, which all look alike. Up-regulated protein abundances are shown in red and decreased proteins levels are in green. B. As the VENN diagram of the identified proteins shows, most of the differentially expressed protein spots lie between the vehicle and α-GalCer-treated WT samples.

When the tested groups were hierarchically distributed, two main clusters were found; one consisting of α-GalCer-treated WT samples, the other containing α-GalCer-treated Jα18^-/-^ and CD1d^-/-^ samples and vehicle-treated WT samples (Fig 4A). In WT mice, α-GalCer enhanced the expression of most of the identified proteins. In another cluster, only one sixth of proteins displayed increased protein production, while most of the proteins were unchanged or their production was decreased when compared to the α-GalCer-treated WT (Fig 4A). In the VENN-diagram, most of the changed protein expressions were observed between vehicle and α-GalCer-treated WT mice, whereas the majority of the protein spots remained unchanged in iNKT cell—deficient mice (Fig 4B). In WT mice, α-GalCer induced proteins function in cell trafficking and modulation, inflammatory response and ion transportation according to the biological annotations in DAVID (Table 2).

**Table 2:** Functional annotation.

Term	Count	%	*p*-value	Proteins
Response to wounding (GO:0009611)	8	27	3,81E-06	P48036, P04186, P32261, O09049, Q921I1, P01027, O35744, Q9WVL7
Inflammatory response (GO:0006954)	6	20	7,14E-05	P04186, O09049, Q921I1, P01027, O35744, Q9WVL7
Carbohydrate catabolic process (GO:0016052)	4	13	5,38E-04	P17182, Q9DBJ1, Q61362, O35744
Acute inflammatory response (GO:0002526)	4	13	5,38E-04	P04186, O09049, Q921I1, P01027
Defence response (GO:0006952)	6	20	1,69E-03	P04186, O09049, Q921I1, P01027, O35744, Q9WVL7
Di-, tri-valent inorganic cation transport (GO:0015674)	4	13	3,88E-03	Q921I1, P29391, P08071, P49945, Q9D7Z6
Chitin catabolic process (GO:0006032)	2	7	1,97E-02	Q61362, O35744
Complement activation, alternative pathway (GO:0006957)	2	7	1,97E-02	P04186, P01027
Homeostatic process (GO:0042592)	5	17	2,70E-02	P99029, Q921I1, P29391, P50404, P08071, P49945
Response to extracellular stimulus (GO:0009991)	3	10	2,84E-02	P04186, P32261, P07724
Cellular homeostasis (GO:0019725)	4	13	2,98E-02	P99029, Q921I1, P29391, P08071, P49945
Cellular cation homeostasis (GO:0030003)	3	10	3,63E-02	Q921I1, P29391, P08071, P49945
Actin cytoskeleton organization (GO:0030036)	3	10	4,22E-02	P62962, P31725, P59999

The most enriched biological functional annotations according to DAVID (GOTERM/BP/FAT). Term refers to the annotation item from the database, and count is the number of proteins found in a given category. Percentage refers to the percent of the identified proteins which are under the annotation, while *p*-value is the EASE (modified Fisher's exact *p*-value) score of the annotation.

### Protein validation

**Fig 5:**
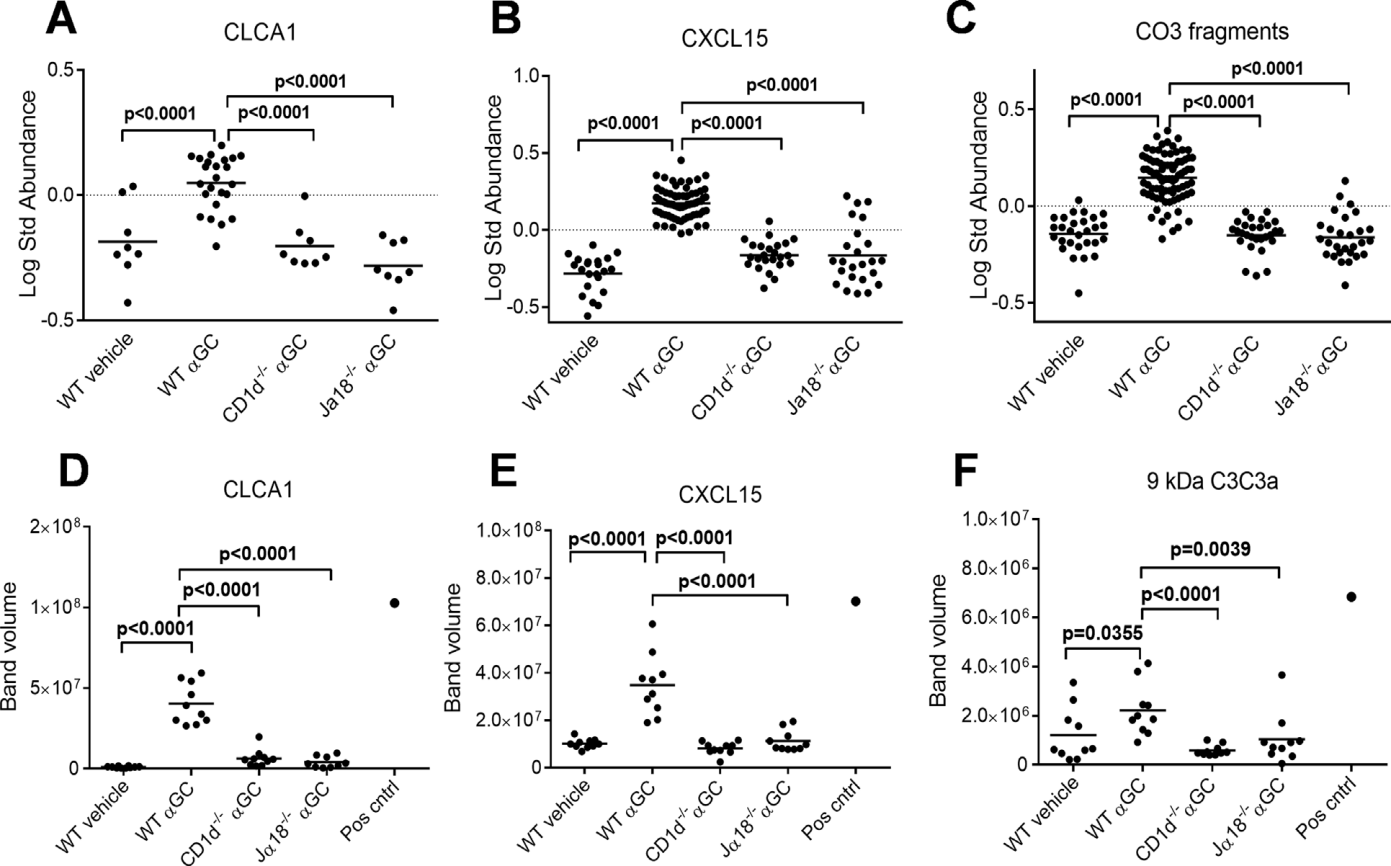
Western blot abundance differences for proteins selected for validation. Protein levels of all identified CLCA1 (A), CXCL15 (B) and complement C3 fragments (C) are shown as dotplots in the study groups as log standard abundance of the gel spots from the DeCyder EDA program. Differences in immunoblot band volumes are shown for CLCA1 (D), CXCL15 (E) and 9 kD C3 fragment called anaphylatoxin C3a (F). Student’s *t*-test p values for significant protein level differences between α-GalCer—treated (αGC) WT mice and other groups are marked. A BALF sample from ovalbumin-sensitized and challenged mouse was used as a positive control for asthma (Pos cntrl).

The 2D-DIGE data analysis and protein identifications were validated for a few selected proteins. The identified peptide spots for CLCA1 (Fig 5A), CXCL15 (Fig 5B) and spliced isoforms of complement C3 (Fig 5C) were combined from 2D gels, and they all showed significantly higher abundances in the WT α-GalCer—treated group when compared to the WT vehicle or CD1d/Jα18 α-GalCer—treated groups. In addition, the mRNA expression of CXCL15 and CLCA1 genes was increased in the α-GalCer—treated WT mice in lung tissue and in BALF cells (S2 Fig). Western blot analyses confirmed that concentrations for CLCA1, CXCL15 and complement C3 cleavage products were significantly higher in α-GalCer—treated WT mice than in the other groups (Fig 5D and 5F). The activation of complement C3 was detected by an antibody which detects several complement C3 subunits, of which 9 kD (C3a) is shown in Fig 5F.

## Discussion

For decades, asthma was considered as an allergic, eosinophilic and Th2-mediated disease, but when Th2-focused therapeutics in human clinical trials seemed to reduce only inflammation, research into these kind of immune-based approaches to treat other types of asthma and especially AHR was retarded [17]. Recent studies have focused on the fact that asthma is not a single entity but instead is a heterogeneic disease with different phenotypes [1], including allergic asthma, steroid-resistant asthma, and asthma induced by exposure to air pollutants, cigarette smoke, diesel exhaust, particles or exercise [18]. These different pathways and phenotypes often coexist and act in synergy, which suggests that more targeted approaches and personalized therapies are needed. Therefore, it is important that new biomarkers are discovered capable of detecting and measuring disease severity and treatment efficacy. In this study, we adopted a proteomic approach to investigate the role of iNKT cells in the development of α-GalCer—induced AHR and airway reactivity, and identified soluble proteins in BALF during AHR in WT as well as in CD1d^-/-^ and in Jα18^-/-^ mice, which lack iNKT cells.

INKT cells are a prerequisite for the development of AHR in Th2-type allergic asthma, but Th2-type T cells alone are not able to induce AHR [6]. INKT cells are also involved in non-Th2-type diseases including ozone-induced (air pollution) and in virus-induced animal models of asthma and AHR [19]. Activated iNKT cells rapidly produce cytokines, including IL-4 and IFN-γ, which activate dendritic cells, macrophages, NK cells, T cell and B cells to induce local inflammation and to drive the development of adaptive immunity. In addition, as a strong activator that may trigger a long-term susceptibility to allergic airway inflammation, α-GalCer has also been shown to be an attenuator of Th2-type responses and AHR in antigen-specific asthma models, depending on the timing, administration route and the *in vivo* model used [20–23]. These results suggest that NKT cells have a crucial role in directing the immunological response. In the present study we used an intranasal administration of α-GalCer to induce AHR and airway inflammation in the WT mice, whereas in CD1d^-/-^ and Jα18^-/-^ mice, α-GalCer’s effects on AHR and inflammation were highly attenuated, as also reported previously [24]. In line with this, also the expression of IL-4, IFN-γ, IL-10 and IL-13 cytokines in lung tissue was abrogated in the iNKT cell—deficient mice.

BALF flushes airways and can therefore provide information about the key components that are produced during the α-GalCer-induced lung inflammation and AHR. According to our proteome data, the α-GalCer-induced iNKT cells had activated several branches of immunity including TLRs, cell phagocytosis, production of mucus, and the complement system. In response to TLR activation, neutrophils, macrophages and epithelial cells produce a protein called neutrophil gelatinase-associated lipocalin (NGAL, also called lipocalin 2) that limits bacterial growth and attracts neutrophils to the site of inflammation [25, 26]. Pulmonary surfactant-associated protein D (SFTPD, former SP-D) recognizes glycoconjugates, inhibits TNF-α production and enhances phagocytosis of inhaled pathogens [27]. Mouse calcium-activated chloride channel regulator 1 (CLCA1 or gob-5) as well as its human homolog is expressed in the goblet cell granules that transport chloride anions into the airway lumen and therefore contributes to the salt and water composition of the secreted mucus [28]. The expression and production of CLCA1 have been reported to increase in airways during asthma in mouse [29, 30] and man [31]. In addition, the versatile complement system and small anaphylatoxins C3a and C5a are believed to promote proallergic effector functions including AHR, eosinophilia, Th2 cytokines and IgE during the development of allergic asthma [32]. These mechanisms involved in this cascade are not fully understood but in our study it seems that the C3 component was not cleaved in those mice that were treated with vehicle or that lacked iNKT cells indicating that α-GalCer clearly activates also the complement system. On the other hand, prevention of the binding of complement C3a to its receptors in mice failed to attenuate AHR in our tests (data not shown).

During the early phase of inflammation, many immune cells, especially neutrophils but also lymphocytes and eosinophils, are recruited into the target site. We observed an accumulation of neutrophils into the BALF at 24 hours after α-GalCer administration, and others have shown that later also eosinophils and T cells are present in mouse BAL [21, 33]. CXCL15 (lungkine) is an important mediator of neutrophil migration from the lung parenchyma into the airspace, and its production is clearly up-regulated in response to a variety of inflammatory stimuli [34]. Although no evident homolog for CXCL15 has been found in humans, a marked increase in neutrophils in human sputum has also been demonstrated during exacerbation incidents in human asthma [35]. Antimicrobial S100-A9 (calgranulin B) dimerizes with S100A8 (calgranulin A) and this acts as a signal for neutrophil and lymphocyte recruitment [36], and in human studies it has been speculated that this could be a biomarker for disease severity in chronic lung diseases [37]. A family of chitinase proteins, including chitinase 3-like 1 (CHI3L1) and chitinase 3-like 3 (CHI3L3 or BRP39 or Ym1) are reported to function as eosinophil chemotactic factors, and CHI3L1 might also induce T lymphocytes, augment adaptive Th2 immunity and stimulate alternative macrophages [38]. In humans, mutations in the *CHI3L1* gene have been shown to associate with some features of asthma [39], and increased CHI3L1 concentrations in lung and serum often correlate with disease severity [38]. Therefore, our results support the use of CHI3L1 as a possible biomarker of inflammation and remodeling in airways as proposed by Konradsen et al. [40].

Different murine airway models show surprisingly similar BALF proteome profile. Zhao et al. identified 28 significantly altered protein spots including lungkine, chitinases Ym1, Ym2, acidic mammalian chitinase, gob-5 and SP-D using ovalbumin-induced Th2-type asthma model in BALB/c mice [35]. Using similar models, proteins Ym1 and SP-D, or Ym1/Ym2, lungkine and SP-D were significantly increased when compared to control groups [41, 42]. In an airway model of oxidative stress, the amounts of complement C3, lipocalin, Ym1, Ym2 and SP-D were also enhanced [43]. In all these models, the apparent lack of identified spots for classic markers of allergic inflammation such IL-5, IL-4 and IL-13 is somewhat surprising but this is most likely due to sensitivity and technical issues while proteomic analyses of biofluids often seem to miss them, even when utilizing a shot-gun method [44]. Nonetheless, our results provide a comprehensive overview of the events in the BALF during the early stages during iNKT-mediated airway inflammation and AHR.

In conclusion, we identified several proteins that contribute to iNKT-dependent AHR via complement activation, leukocyte chemotaxis and airway mucus production. Based on these data, we propose that the combination of CXCL15, S100-A9, CHI3L1/3, SFTPD and CLCA1 would be biomarker candidates for the AHR phenotype. Further studies will be needed to assess the specificity and sensitivity of these biomarkers in patients with AHR and airway inflammation. Furthermore, an iNKT cell-specific antagonists could open new avenues in the therapeutics of certain type of allergic inflammatory responses.

## Supporting Information

S1 Fig BALF cytospin and H&E staining of lungs. A representative microscope image of MGG-stained, cytospinned BALF samples from WT mice which were treated with vehicle (DMSO) or with α-GalCer (αGC). Immunohistochemical staining was performed on lung sections from CD1^-/-^ and Jα18^-/-^ mice after vehicle or α-GalCer-treatment (αGC). Only one sample is shown as an example and there were 8 mice/group.
(TIF)

S2 Fig RT-pcr analysis of lung and BALF cells. Messenger-RNA expressions of validated CXCL15 and CLCA1 proteins by RT-PCR from lung specimen (A) and from BALF (B) cells. In both cases, samples are collected 24 hours after intranasal challenge.
(TIF)
