# Supplementary figures and images for: Invariant Natural Killer T Cells Play a Role in Chemotaxis, Complement Activation and Mucus Production in a Mouse Model of Airway Hyperreactivity and Inflammation
Source: PLoS One. 2015 Jun 12;10(6):e0129446. doi: 10.1371/journal.pone.0129446 (PMC4466557; doi:10.1371/journal.pone.0129446)

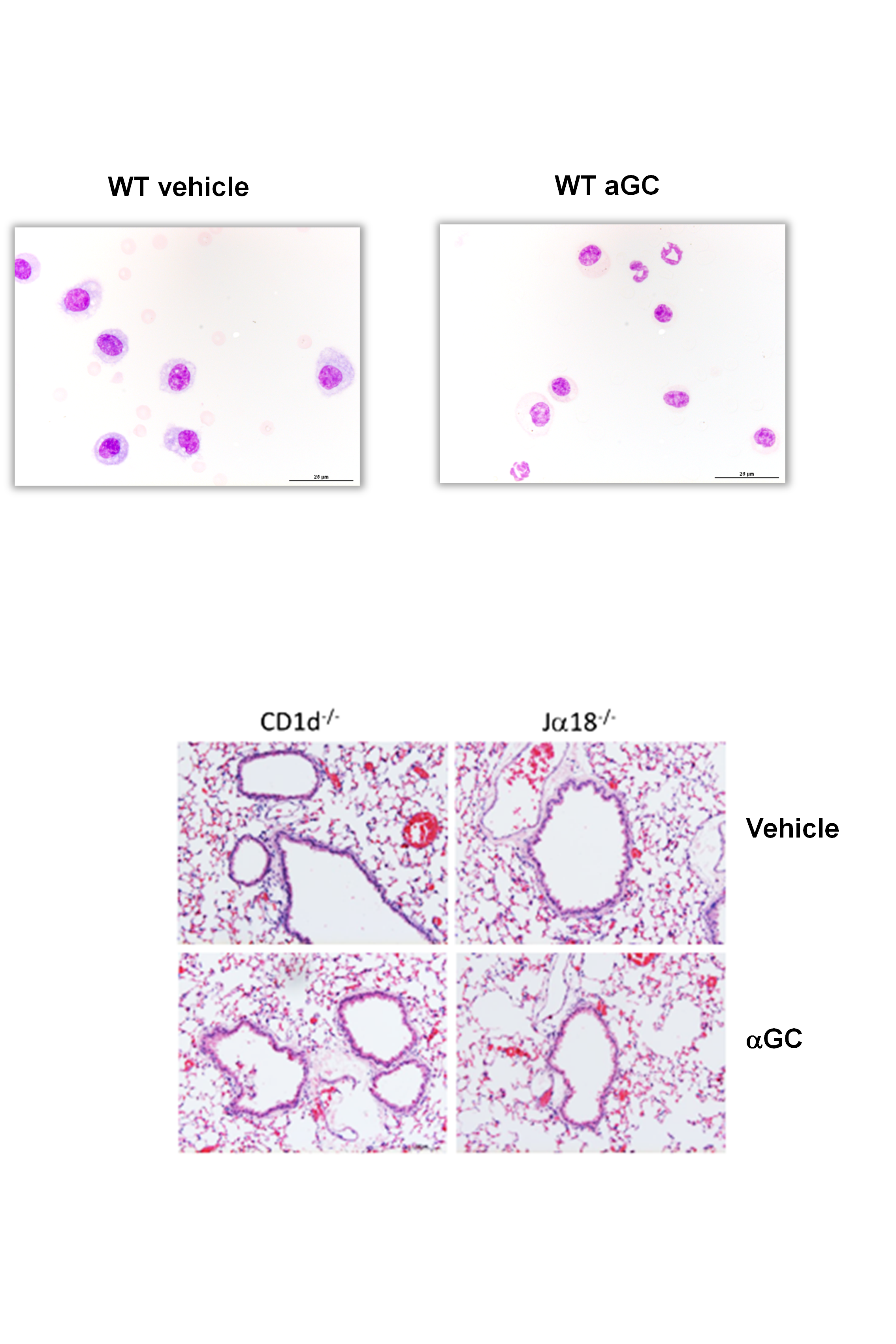

Supplement: S1 Fig — A representative microscope image of MGG-stained, cytospinned BALF samples from WT mice which were treated with vehicle (DMSO) or with α-GalCer (αGC). Immunohistochemical staining was performed on lung sections from CD1-/- and Jα18-/- mice after vehicle or α-GalCer-treatment (αGC). Only one sample is shown as an example and there were 8 mice/group. (TIF) [file pone.0129446.s001.tif]

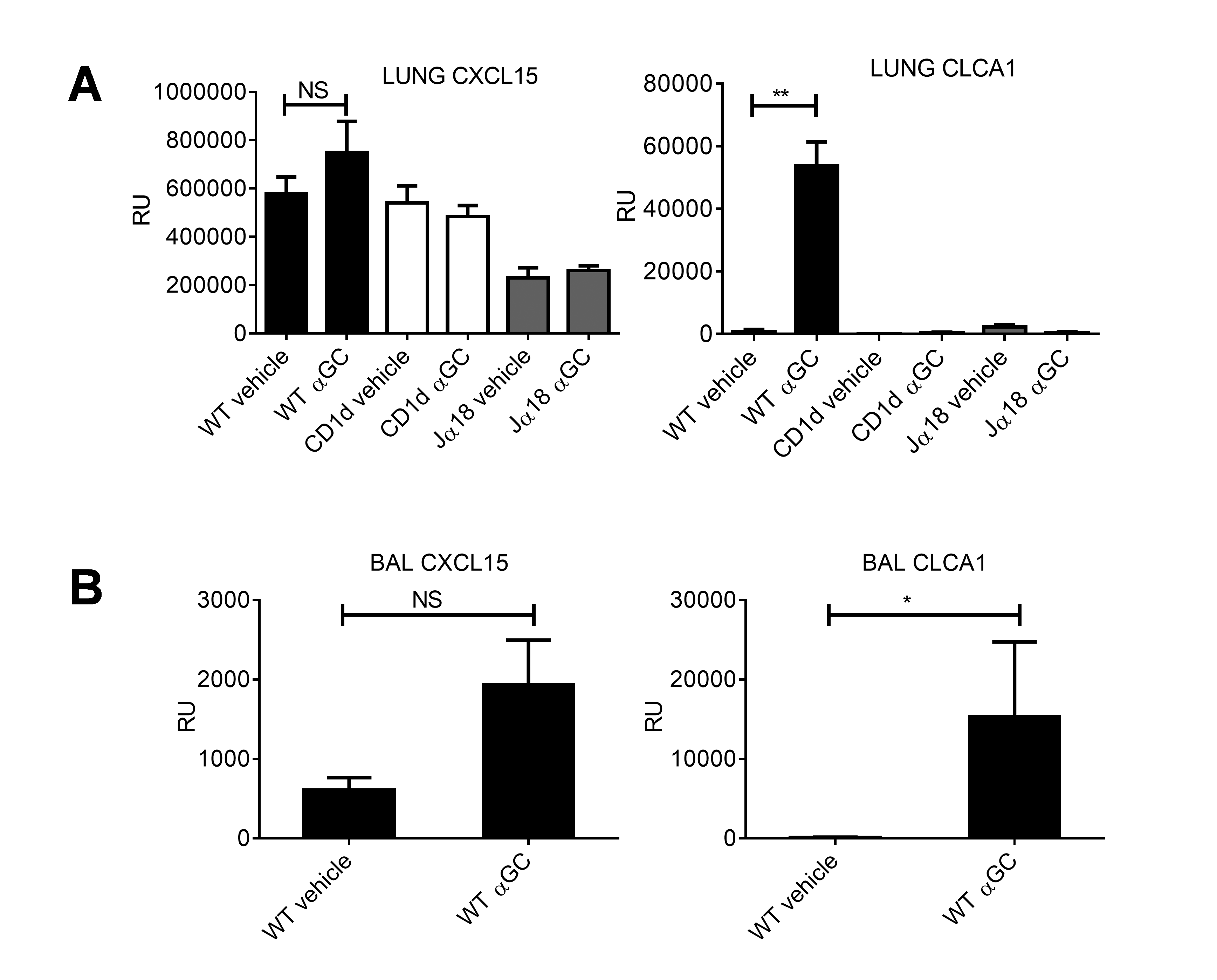

Supplement: S2 Fig — Messenger-RNA expressions of validated CXCL15 and CLCA1 proteins by RT-PCR from lung specimen (A) and from BALF (B) cells. In both cases, samples are collected 24 hours after intranasal challenge. (TIF) [file pone.0129446.s002.tif]
